# A 24 GHz CMOS Direct-Conversion RF Receiver with I/Q Mismatch Calibration for Radar Sensor Applications

**DOI:** 10.3390/s22218246

**Published:** 2022-10-27

**Authors:** Yongho Lee, Soyeon Kim, Hyunchol Shin

**Affiliations:** 1 Department of Electronic Convergence Engineering, Kwangwoon University, Seoul 01897, Korea; 2 Samsung Electronics Co., Ltd., Suwon 16677, Korea

**Keywords:** RF receiver, direct conversion, I/Q mismatch calibration, 24 GHz, radar, millimeter-wave, CMOS

## Abstract

A 24 GHz millimeter-wave direct-conversion radio-frequency (RF) receiver with wide-range and precise I/Q mismatch calibration is designed in 65 nm CMOS technology for radar sensor applications. The CMOS RF receiver is based on a quadrature direct-conversion architecture. Analytic relations are derived to clearly exhibit the individual contributions of the I/Q amplitude and phase mismatches to the image-rejection ratio (IRR) degradation, which provides a useful design guide for determining the range and resolution of the I/Q mismatch calibration circuit. The designed CMOS RF receiver comprises a low-noise amplifier, quadrature down-conversion mixer, baseband amplifier, and quadrature LO generator. Controlling the individual gate bias voltages of the switching FETs in the down-conversion mixer having a resistive load is found to induce significant changes at the amplitude and phase of the output signal. In the calibration process, the mixer gate bias tuning is first performed for the amplitude mismatch calibration, and the remaining phase mismatch is then calibrated out by the varactor capacitance tuning at the LO buffer’s LC load. Implemented in 65 nm CMOS process, the RF receiver achieves 31.5 dB power gain, −35.2 dBm input-referred 1 dB compression power, and 4.8–7.1 dB noise figure across 22.5–26.1 GHz band, while dissipating 106.2 mA from a 1.2 V supply. The effectiveness of the proposed I/Q mismatch calibration is successfully verified by observing that the amplitude and phase mismatches are improved from 1.0–1.5 dB to 0.02–0.19 dB, and from 10.8–23.8 to 1.1–3.2 degrees, respectively.

## 1. Introduction

A 24 GHz millimeter-wave band is widely used for short-range radar sensor systems targeting automotive, industrial, healthcare, and various sensory applications [1]. This band is also adopted for the fifth-generation (5G) wireless communication as it is assigned to one of the frequency range 2 (FR2) band [2]. With such wide and popular adoptions in various wireless sensor and communication systems, CMOS implementation of 24 GHz RF transceiver-integrated circuits (IC) is highly required for realizing low-power small-form-factor single-chip radio systems. Various CMOS design results addressing the relevant design issues of the RF transmitter [3], RF receiver [4], and LO generation [5] have been reported in the literature. As part of such design efforts, this work presents a design of a 24 GHz CMOS RF receiver with a wide-range and precise I/Q mismatch calibration technique.

Quadrature down-conversion architecture is preferably chosen for a millimeter-wave CMOS RF receiver due to the low hardware complexity, low power dissipation, and flexible interface with an accompanying digital baseband processor. We can find that the architecture has been adopted by a variety of 20–60 GHz-band RF receivers developed for a variety of target applications, for example, the 20–30 GHz FMCW and UWB radar applications [6,7,8,9,10], 28 GHz 5G applications [11], and 60 GHz wireless local-area network (also known as WiGig) applications [12,13]. The quadrature down-conversion architecture is also advantageous considering that it is seamlessly combinable with a millimeter-wave beamforming circuit, either in active type [11] or in passive type [14].

The I/Q signal amplitude and phase mismatch is one of the critical performance metrics in the quadrature down-conversion RF receiver. When the baseband signal is formed by a quadrature amplitude modulation (QAM) and has an asymmetric spectrum profile, it will be corrupted when the I/Q imbalance is not sufficiently minimized, and the image component is not well rejected. Usually, the I/Q mismatch calibration is primarily done in the digital signal processing domain [15]. However, the digital-domain calibration alone usually cannot cover the severe mismatches induced by the accompanying CMOS RF receiver. Hence, an analog-domain calibration is needed together in order to acquire the best I/Q balance and satisfactory image rejection ratio (IRR). Moreover, the analog-domain calibration is also needed if the quadrature down-conversion scheme adopts a low IF frequency plan, because in which the image-rejection is typically done in the analog domain by using a complex bandpass filter such as [16]. In spite of the importance of the I/Q mismatch calibration issue, interestingly, we have found that it has not been often addressed in the previous millimeter-wave CMOS receiver designs for the radar applications [6,8,9,10].

In this work, we first rearrange the widely known relation of IRR versus I/Q mismatch, and newly derive analytic expressions to provide a useful insight on how much the amplitude and phase mismatches individually contribute to the IRR degradation. The newly derived expressions are used as a design guide to the I/Q mismatch calibration circuit design in terms of its range and resolution. Next, we present a design of a 24 GHz CMOS quadrature down-conversion zero-IF RF receiver IC. In author’s previous millimeter-wave up-conversion mixer design having an inductive load [2], the mixer’s gate bias control was found to induce only the amplitude change. However, in this work, we observe significantly different responses when the same technique is applied to a down-conversion mixer having a resistive load. This phenomenon is thoroughly examined and addressed to achieve an optimal I/Q mismatch calibration circuit in the 24 GHz receiver. Measured results show that the proposed I/Q mismatch calibration technique successfully minimize the I/Q signal imbalances in the fabricated CMOS RF receiver IC.

## 2. Analysis of Image-Rejection Ratio (IRR) with Respect to I/Q Mismatch

Let us assume that the down-converted baseband I/Q signals $x_{BB,i}\left(t\right)$ and $x_{BB,q}\left(t\right)$ having the amplitude and phase mismatches of $a_{e}$ and $\theta_{e}$ are expressed as

(1) $$x_{BB,i}\left(t\right)=A_{sig}\cos\left(\omega_{BB}t\right)$$

(2) $$x_{BB,q}\left(t\right)=A_{sig}\left(1+a_{e}\right)\sin\left(\omega_{BB}t+\theta_{e}\right)$$

where $A_{sig}$ and $\omega_{BB}$ are the amplitude and frequency of the baseband signal. It is well known that the IRR is given by [2,13]

(3) $$\mathrm{IRR}=\frac{1+{\left(1+a_{e}\right)}^{2}+2\left(1+a_{e}\right)\cos\left(\theta_{e}\right)}{1+{\left(1+a_{e}\right)}^{2}-2\left(1+a_{e}\right)\cos\left(\theta_{e}\right)}\;\;\;\;\;\;\;\;\;\;\;\;$$

**Figure 1:**
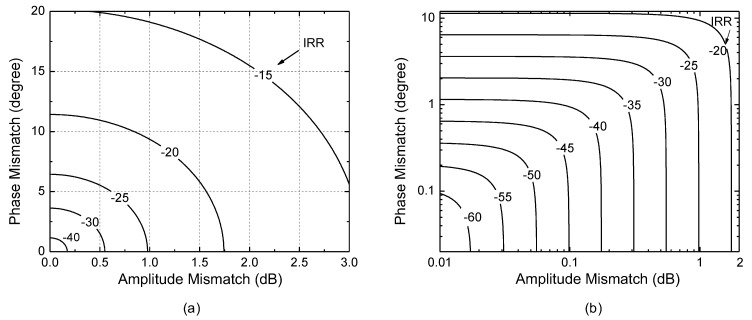
Theoretical image rejection ratio (IRR) with respect to the amplitude and phase mismatches. (**a**) *x*- and *y*-axis in linear scale; (**b**) *x*- and *y*-axis in logarithmic scale.

Figure 1 shows the two-dimensional contour plot of IRR with respect to the amplitude and phase mismatches. Note that the amplitude mismatch used for the *x*-axis is not $a_{e}$, but $\left(1+a_{e}\right)$ so that the no amplitude mismatch condition corresponds to 0 dB, and the amplitude mismatch expressed in dB is obtained by $20\times \log\left(1+a_{e}\right)$. For the sake of clarity in the following discussion, let us denote the amplitude mismatch in dB by $a_{e,dB}$, which corresponds to $a_{e,dB}=20\times \log\left(1+a_{e}\right)$.

Figure 1a,b are drawn by using the same data set, but Figure 1a is drawn with *x*- and *y*-axis in linear scale, whereas Figure 1b is drawn with *x*- and *y*-axis in logarithmic scale. The logarithmic scale plot of Figure 1b better exhibits the relationship in the low IRR (<−40 dB) region. In addition, the logarithmic scale plot newly exhibits an almost logarithmic proportionality between the IRR and mismatches. From Figure 1b, we can loosely state that the IRR in dB is almost linearly proportional to the logarithmic values of the phase mismatch in degree ($\theta_{e}$) and the amplitude mismatch in dB $\left(a_{e,dB}\right)$. Let us denote the phase mismatch in degree by $\theta_{e,deg}$ such that $\theta_{e,deg}=\frac{\pi }{180}\theta_{e}$. Now, we want to analyze this proportionality with respect to the amplitude and phase mismatches, respectively. First, the IRR induced by the amplitude mismatch alone can be found by letting $\theta_{e}=0$ in (3). Then, the IRR of (3) is simplified as

(4) $$\mathrm{IRR}={\left(\frac{2+a_{e}}{a_{e}}\right)}^{2}.\;\;\;\;\;\;\;\;\;\;\;$$

In (4), let us reply $a_{e}$ by ($10^{\frac{a_{e,dB}}{20}}-1)$, and the exponential term $10^{\frac{a_{e,dB}}{20}}$ is approximated by its power series, in which the second- and higher-order terms of the power series are neglected because $a_{e,dB}$ is much less than 20 dB in practice. Then, $a_{e}$ can be approximated to $\frac{ln\left(10\right)}{20}\times a_{e,dB}$. As a result, (4) can be written as

(5) $$\mathrm{IRR}={\left(\frac{k+a_{e,dB}}{a_{e,dB}}\right)}^{2},\;\;\;\;\;\;where\;k=\frac{40}{\ln\left(10\right)}\;\;\;\;\;\;\;\;\;\;\;\;\;\;\;\;\;\;\;\;\;\;\;\;\;\;\;\;\;\;\;$$

Since typical $a_{e,dB}\;$ is much less than *k* = 17.4 dB, (5) can be further simplified to $\mathrm{IRR}\;\left(\mathrm{dB}\right)=24.8-20\cdot \log(a_{e,dB})$. For example, when $a_{e,dB}$ varies from 0.1 to 1 dB, (5) gives the IRR varies from 44.8 dB to 24.8 dB, which agrees well with Figure 1b.

The IRR induced by the phase mismatch can be found by letting $a_{e}=0$ in (3). Then, the IRR of (3) is simplified as

(6) $$\mathrm{IRR}=\frac{1+\cos\left(\theta_{e}\right)}{1-\cos\left(\theta_{e}\right)}\;\;\;\;$$

Now, an interesting relationship is derived by equating (5) and (6). By solving (5) = (6), we can find the equivalent amount of $a_{e,dB}$ and $\theta_{e}$ that leads to the same amount of IRR. From (5) = (6), we can get

(7) $$a_{e,dB}=k\frac{1-\cos\left(\theta_{e}\right)+\sqrt{1-\cos^{2}\left(\theta_{e}\right)}}{2\cos\left(\theta_{e}\right)}\;\;\;\;\;\;\;\;\;\;$$

By using the power series of $\cos\left(\theta_{e}\right)\;$ with the second- and higher-order terms neglected since $\theta_{e}\ll 1$ in practice, $\cos\left(\theta_{e}\right)\;$ can be approximated to $1-\frac{\theta_{e}^{2}}{2}$. Additionally, by replacing $\theta_{e}$ by $\theta_{e,deg}$, we can obtain the following relation,

(8) $$\theta_{e,deg}=6.6\cdot a_{e,dB}\;\;\;\;$$

(8) implies that 1 dB amplitude mismatch induces the same amount of IRR degradation with 6.6-degree phase mismatch. It also implies that calibration range and resolution for the phase mismatch in degree should be 6.6 times larger than that for the amplitude mismatch in dB. For example, let us assume that the initial IRR before calibration is as worse as −20 dB, and we want to calibrate it to better than −50 dB, we will need the amplitude mismatch calibration to be conducted over a range of 2 dB with a resolution of 0.05 dB, and the accompanying phase mismatch calibration to be conducted over a range of 13.2 degrees with a resolution of 0.33 degrees.

## 3. Circuit Design

### 3.1. Architecture

**Figure 2:**
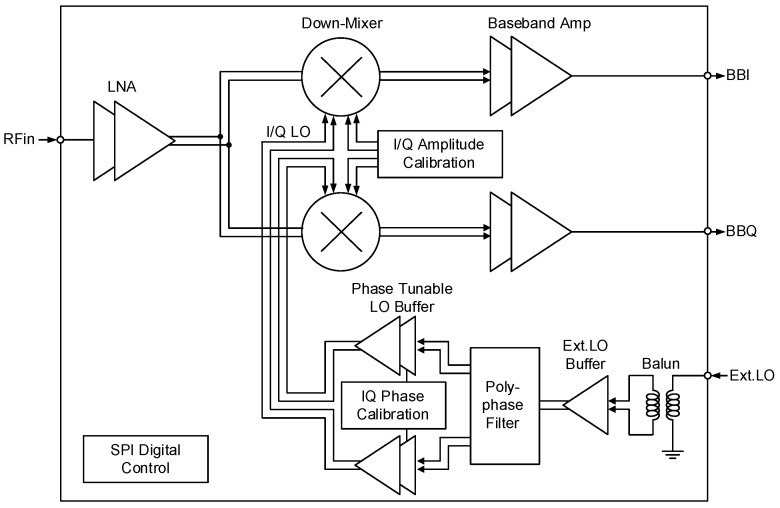
The 24 GHz direct-conversion RF receiver architecture.

Figure 2 shows the architecture of the 24 GHz CMOS RF receiver. It is based on the quadrature direct down-conversion architecture. This architecture can have either zero-IF or low-IF because that the image rejection operation can be subsequently conducted using the down-converted I/Q signals either in the analog or digital domain. The RF receiver comprises a low-noise amplifier (LNA), quadrature down-conversion mixer, and baseband amplifier (BBA). The single-ended RF input is converted to differential at the LNA, and subsequently processed in differential. The final output signal is converted back to single-ended at the BBA to provide the quadrature baseband output signal BBI and BBQ, which facilitates the chip test and characterization. For the quadrature local oscillator (LO) signal generation, the single-ended external LO signal is converted to differential by using an on-chip transformer balun, and subsequently to differential I/Q LO signals by using a single-stage RC-CR polyphase filter. The subsequent LO buffers boost the LO swing sufficiently large enough to drive the down-conversion mixer. The serial peripheral interface is used to control the digital states of the chip. Note that one of the author’s prior work [17] also adopted the same architecture for a 28 GHz CMOS RF receiver, but it presented only preliminary transistor-level simulation results without any implementation or measurements results in CMOS.

The I/Q mismatch calibration is realized through two tuning circuits, one at the mixer and the other at the LO buffer, as shown in Figure 2. The same approach was shown effective for author’s previous 28 GHz direct-conversion RF transmitter [2]. Since this 24 GHz RF receiver are based on the same direct-conversion architecture with the author’s previous RF transmitter, when we employ a quadrature Gilbert-cell active mixer and also the same LO generation scheme, it is a very reasonable anticipation that the same calibration technique that is already proven effective for the direct-conversion RF transmitter should be equally effective for this direct-conversion RF receiver. However, we have found significantly different behaviors for the mixer tuning circuits between the up- and down-conversion mixers. Let us describe the differences and considerations related to the mixer tuning circuit.

### 3.2. Down-Conversion Mixer and I/Q Calibration Circuit Design

**Figure 3:**
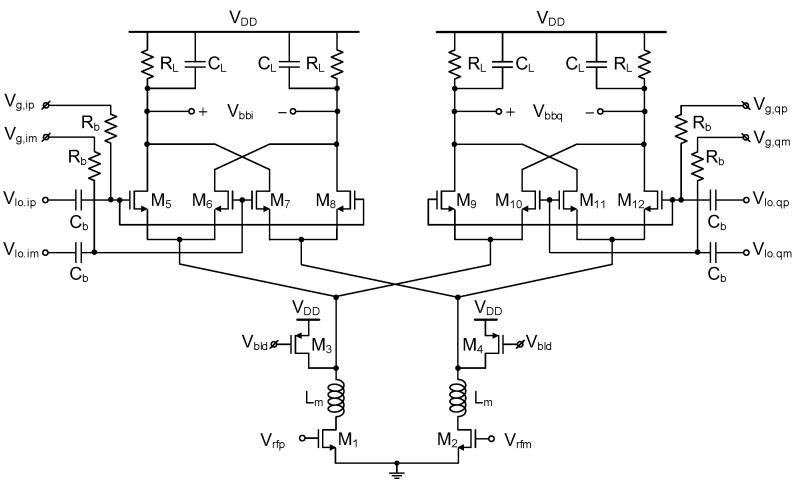
Quadrature down-conversion mixer schematic.

The circuit schematic of the quadrature down-conversion mixer is shown in Figure 3. It has a double-balanced Gilbert-cell structure. The RF input signal V_rfp_, V_rfm_ come from the preceding LNA, and the baseband output signal V_bbi_, V_bbq_ are transferred to the subsequent BBA. The transconductance (g_m_) stage FETs M_1–2_ has a total gate width of 64 μm with 11 mA bias current. The switching stage FETs M_5–12_ have a total gate width of 80 μm. The load resistor R_L_ and capacitor C_L_ are 350 Ω and 290 fF, respectively, whose low-pass corner frequency is 1.6 GHz, which sufficiently covers the desired channel bandwidth of 700 MHz while suppressing the higher frequency components. By adding two shunt FETs M_3,4_ (total gate width of 32 μm), part of the g_m_-stage bias current is bled to the supply and only the remaining 2.8 mA flows through the mixer. Thus, the IR drop in R_L_ is minimized to 0.5 V. The current bleeding technique reduces the current-resistance (IR) drop in R_L_, which in turn improves the swing headroom and output linearity. The reduction of the bias current through the switching FETs also leads to more efficient and fast switching, thus improving the gain and noise figure performance.

**Figure 4:**
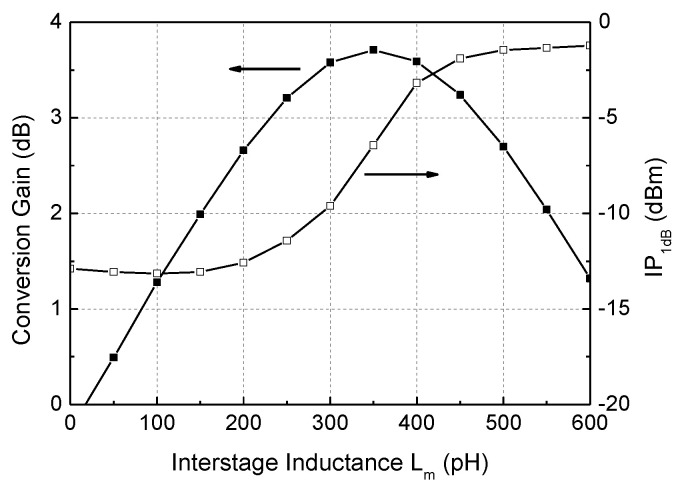
Effects of the interstage inductance L_m_ on the mixer performance.

The interstage inductor L_m_ is placed between the g_m_-stage M_1–2_ and switching stage M_5–12_. It resonates with the parasitic capacitances existing at the node, leading to the gain, linearity, and noise figure improvements. Due to the known advantages, this technique is often found in the millimeter-wave down-conversion mixer [9,11] and LNA [18]. Figure 4 shows the simulation results of the conversion gain and input-referred 1 dB compression point (IP_1dB_) of the mixer with respect to L_m_. In this design, 350 pH is chosen to achieve an optimal performances of the conversion gain of +3.5 dB and IP_1dB_ of −6.5 dBm.

As shown in Figure 3, the differential I/Q LO signals V_lo,ip_, V_lo,im_, V_lo,qp_, V_lo,qm_ are fed to the switching FETs M_5–12_ via the ac-coupling capacitor C_b_ of 1 pF. Their independent bias voltages V_g,ip_, V_g,im_, V_g,qp_, V_g,qm_ are applied via the ac-blocking resistors R_b_ of 1 kΩ. In the previous RF up-conversion mixer [2], authors simply demonstrated that adjusting the mixer’s gate bias voltages could effectively tune the I/Q amplitudes. Nonetheless, we have found that the same technique here shows significant different behavior for the RF down-conversion mixer. Comparative simulation investigations are carried out to reveal the differences.

**Figure 5:**
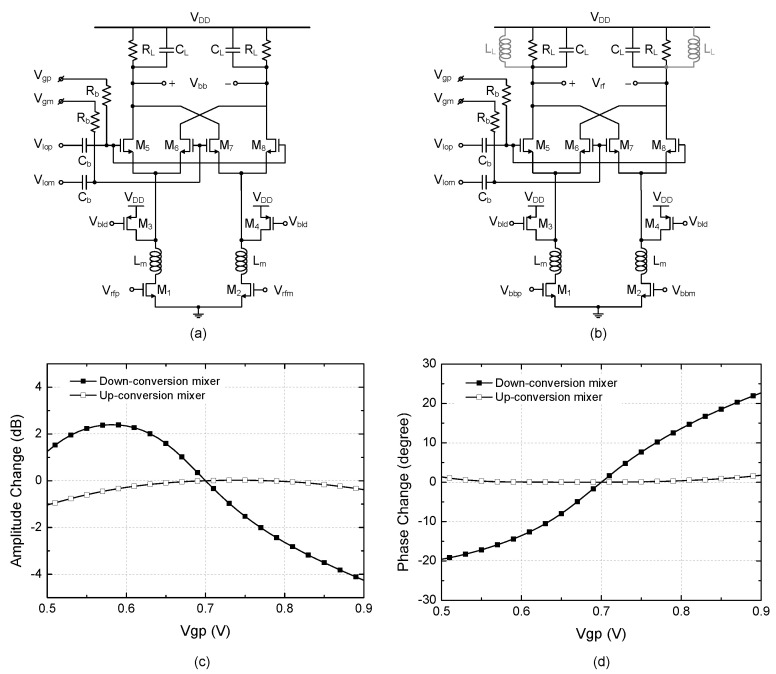
Effects of the gate bias control on the output phase and amplitude. (**a**) Down-conversion mixer with R_L_C_L_ load; (**b**) Up-conversion mixer with L_L_R_L_C_L_ load; (**c**) Amplitude change; (**d**) Phase change.

Figure 5a is only the single-path circuit out of the original dual-path quadrature down-conversion mixer. It is enough to examine only the single-path circuit because the dual-path circuit cores produce identical responses. For the simulation, we apply the RF input with a frequency of 24 GHz and power of −40 dBm, the LO signal with a frequency of 23.5 GHz and power of 0 dBm. The resulting baseband signal at 500 MHz is probed at the output. In order to examine the different tuning behaviors in an up-conversion mixer, Figure 5b is also simulated for comparison. For the up-conversion mixer, a shunt inductor L_L_ of 120 pH is added in parallel to R_L_C_L_ so that it can resonate with the load capacitance. We apply the baseband and LO signals at 500 MHz and 23.5 GHz, respectively, and the RF signal of 24 GHz is probed at the output.

For the down- and up-conversion mixers, the relative changes of the amplitude and phase at the output signal are plotted in Figure 5c,d, respectively, when V_gp_ is swept from 0.5 V to 0.9 V, while V_gm_ is fixed at 0.7 V. It is interesting to observe that the down-conversion mixer shows more dramatic change of the amplitude and phase than the up-conversion mixer. The down-conversion mixer shows a relatively large amplitude change of +2.38–−4.25 dB, while the up-conversion mixer shows much smaller amplitude change of −1.05 dB. Similarly, the much larger phase change of −19.5–+22.7 degrees is observed at the down-conversion mixer, while much smaller change of only 0–+1.7 degrees is observed at the up-conversion mixer. Hence, we can conclude that the amplitude and phase changes induced by the gate bias control technique is much more sensitive and dramatic in the down-conversion mixer than in the up-conversion mixer.

**Figure 6:**
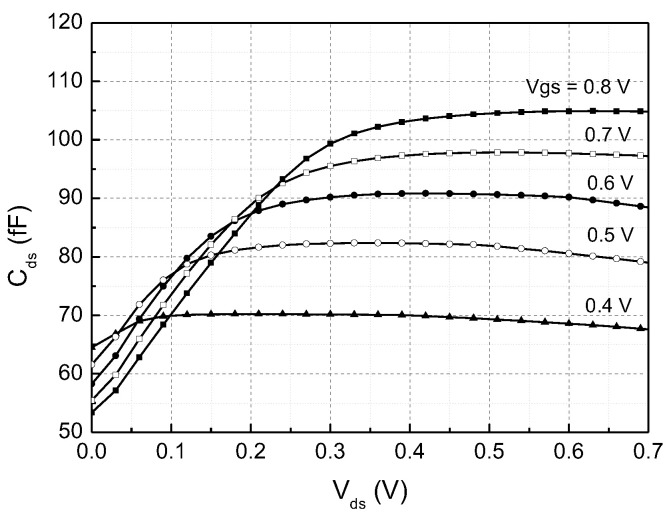
Drain-to-source capacitance C_ds_ with respect to drain-to-source voltage V_ds_.

The different tuning behaviors are attributed to the different dc bias conditions of the switch FETs. In the down-conversion mixer that has the RC load, the drain-source voltage V_ds_ of the switch FETs M_5-8_ is set to relatively small 0.1–0.4 V due the voltage drop via R_L_. However, in the up-conversion mixer with the RLC load, V_ds_ is set as high as 0.5–0.7 V because the load inductor L_L_ eliminates the IR drop. This different V_ds_ leads to significant difference of C_ds_ of the switch FETs. To confirm this, we have simulated C_ds_ against V_ds_ for an FET identical with one of M_5–8_ of Figure 5a,b. As can be seen in Figure 6, C_ds_ shows much sensitive dependence with respect to V_ds_ at low V_ds_ region, while it remains rather constant at high V_ds_ region. Since the effective V_ds_ should vary as the dc gate bias voltage is controlled, this sensitive variation of C_ds_ with respect to V_ds_ will lead to much more phase variation at the output. This effect can be also understood by recognizing that the switch FET’s parasitic admittance affect the output phase and amplitude at the Gilbert-cell active mixer that was proven by Shahani et al. [19].

In addition to this C_ds_ effect, the gate bias control also alter the effective duty cycle of the LO signal arriving at the switching FETs, and also alter the transconductance and output resistance of the g_m_-stage FETs M_1,2_ and M_7,8_, which result in the amplitude change at the output.

**Figure 7:**
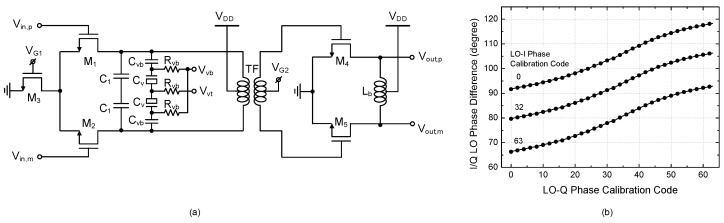
Phase-tunable LO buffer. (**a**) Schematic; (**b**) Phase tuning simulation result.

Since the mixer gate bias control induces the significant amplitude and phase changes at the down-conversion mixer, the phase calibration by the phase-tunable LO buffer must be designed properly to cover not only the inherent phase mismatch but also the residual phase change. Figure 7a shows the phase-tunable LO buffer schematic. The LO phase tuning is performed by tuning the varactor diode at the load. For precise calibration, the varactor tuning voltage is generated by a 6-bit R-2R voltage digital-to-analog converter (VDAC). From simulations considering the layout parasitics and 3-dimensional full-wave electromagnetic field effects, we have found that the I/Q phase tuning covers −23.7–+28.3 degrees with a 0.8-degree resolution. It should be pointed out that this technique provides wider, finer, and more robust phase tuning capability than various conventional methods such as capacitance tuning in the RC-CR filter [20], floating capacitance tuning in the buffer’s load [21], buffer bias current tuning [12], or the tank capacitance offsetting in quadrature VCO [11,13].

### 3.3. LNA and Output Buffer

**Figure 8:**
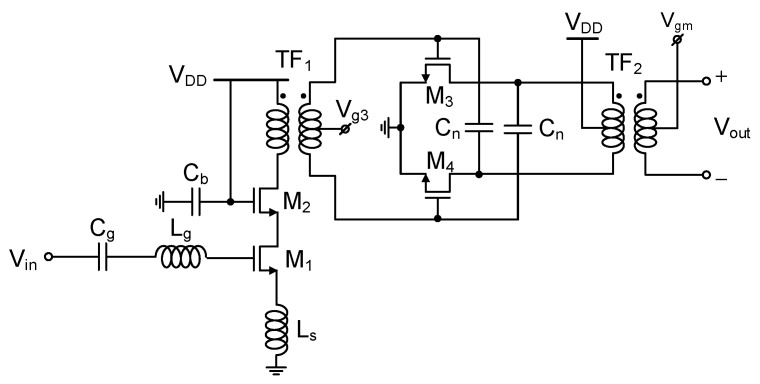
Low-noise amplifier (LNA).

The LNA schematic is shown in Figure 8. It comprises two stages. The first stage is a cascode M_1,2_ with a transformer balun TF_1_ at the load for single-to-differential conversion. The second stage is a pseudo-differential pair M_3,4_ with a transformer load TF_2_ coupled to the following mixer. The center tap of the secondary turn of TF_2_ is used to feed the gate bias voltage for the mixer’s g_m_ stage of Figure 3. For millimeter-wave LNAs, single-ended circuit structure should be more advantageous for achieving a lower noise figure than a differential structure [11,13]. However, making the entire LNA in a single-ended structure could severely degrade the common-mode noise immunity. Thus, we choose to convert the single-ended structure of the first stage to differential at the second stage as in [22,23]. The transformer balun TF_1_ is adopted for that purpose.

The LNA’s first stage dissipates 14 mA, and the gate widths of M_1_ and M_2_ are 80 μm and 64 μm, respectively. The series C_g_ and L_g_ of 475 fF and 450 pH are added for the input impedance matching and dc blocking. The degeneration inductor L_s_ of 74 pH simultaneously improves the gain and noise performance. The bypass capacitor C_b_ of 2 pF improves the ac ground of M_2_ gate, leading to better immunity to the supply noise. The second stage dissipates 19.5 mA, and the gate width of M_3,4_ is 50 μm. The neutralized capacitor C_n_ of 16 fF in a metal-oxide-metal structure improves the stability and gain [24].

**Figure 9:**
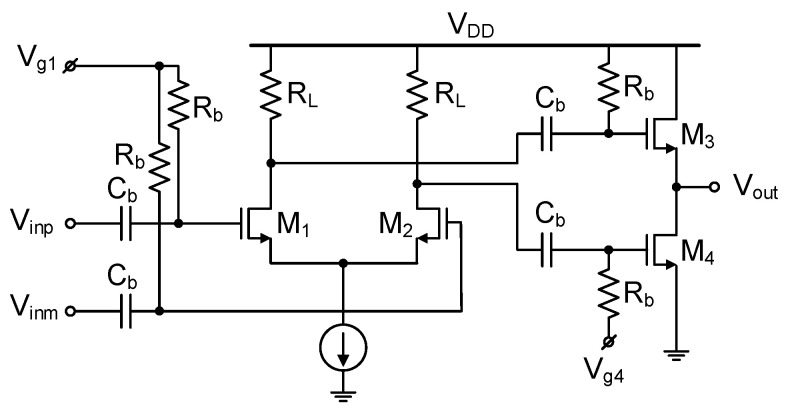
Baseband amplifier (BBA).

The BBA comprises two stages as shown in Figure 9. The first stage is a differential pair M_1,2_ having the gate width of 80 μm. The load R_L_ is 110 Ω. It dissipates 6.1 mA to give the voltage gain of 8 dB. The second stage is designed to sufficiently drive an external 50 Ω load in an impedance matched condition so that the high-speed baseband output signal can be properly tested by an external test equipment. It combines a source follower stage M_3_ and a common-source stage M_4_ to convert the differential to a single-ended final output signal V_out_ [25]. The gate bias voltage V_g4_ for the common-source stage M_4_ is tuned to maximize the conversion gain and minimize the even-order harmonic distortion components at the output.

## 4. Implementation Results

**Figure 10:**
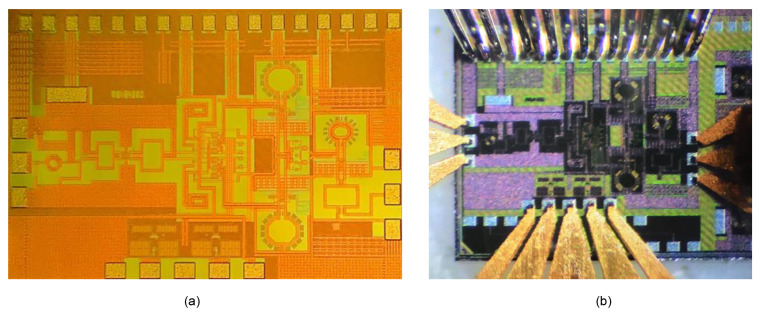
(**a**) Chip micrograph; (**b**) On-wafer probing measurement setup.

The 24 GHz RF receiver IC of Figure 2 is fabricated in a 65 nm RF CMOS process. A micrograph of the fabricated chip is shown in Figure 10a. The die size is 1.71 × 1.15 mm^2^ including the pad frame. The dc pads are located at the top, and the signal pads are at the side and bottom of the die. The fabricated chip is tested through the on-wafer probing as shown in Figure 10b. The 24 GHz RF input signal is fed by an ground–signal–ground (G-S-G) probe at the left, the LO signal is fed by an G-S-G probe at the right, and the baseband I/Q output signal is taken by the G-S-G-S-G probe at the bottom. The dc and low-frequency serial-peripheral-interface (SPI) signals are supplied through the 17-pin probe from the top. Although not shown in Figure 10b, we also have performed the test by mounting the chip on a printed circuit board (PCB) where the 17 top-side pins are wire-bonded and the rest signals of RF, LO, and baseband are probed on the wafer. Such on-PCB measurement, compared to the full on-wafer measurement, shows more stable and reliable results due to more solid and wide ground plane provided by the PCB. A single 1.2 V supply voltage is used for the measurements.

**Figure 11:**
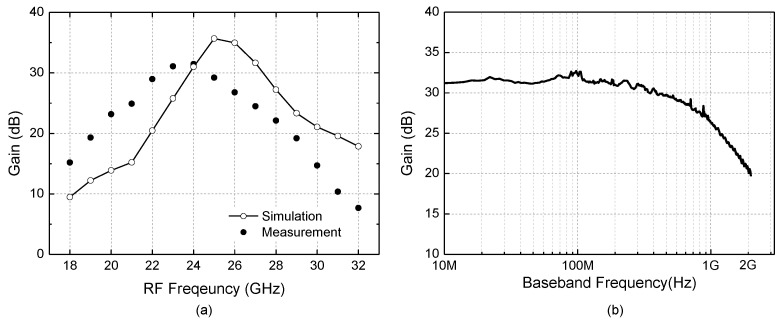
Frequency response characteristics. (**a**) Gain versus RF frequency; (**b**) Gain versus baseband frequency.

Figure 11a shows the measured and simulated power gains against the RF frequency. The RF and LO frequencies are swept from 18 to 32 GHz with 100 MHz spacing fixed so that the baseband frequency remains at 100 MHz. The measured peak gain of +32.4 dB appears at 24 GHz, whereas the simulation results show a slightly higher gain of +35.7 dB at a slightly higher frequency of 25 GHz. The 3 dB operating band is 22.5–26.1 GHz. Figure 11b shows the measured baseband bandwidth. The LO frequency is fixed at 24 GHz, and the RF frequency is swept from 24.1 to 26 GHz. The 3 dB channel bandwidth is found to be 700 MHz.

**Figure 12:**
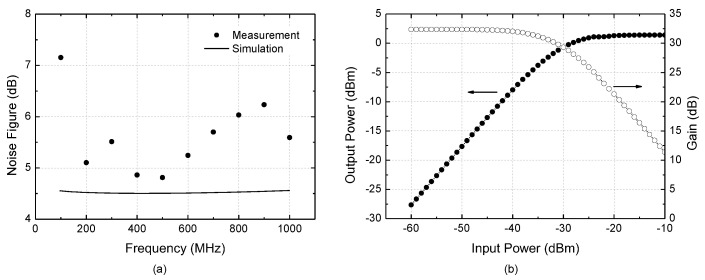
Measured results. (**a**) Noise figure; (**b**) Input-to-output power transfer characteristic.

Figure 12a shows the measured and simulated noise figure. For the noise figure measurement, the total output noise power P_n,out_ is first measured by using a spectrum analyzer with the input port terminated by 50 Ω. Then, the noise figure is calculated by (P_n,out_ + 174 dBm/Hz − G_P_) in dBm, where G_P_ is the power gain in dB. This method is convenient because it does not need a noise source or noise figure meter while ensuring sufficient accuracy in a condition that the noise floor level of the used spectrum analyzer is much lower than P_n,out_. The measured noise figure at 100–1000 MHz is 4.8–7.1 dB, whereas the simulated noise figure is about 4.5 dB. The disagreement between the simulated and measured noise figures would be attributed to the uncertainties of the measurement equipments, connectors, cables, and their setup. Figure 12b shows the measured input-to-output power transfer characteristic. The input- and output-referred 1 dB compression powers IP_1dB_ and OP_1dB_ are −35.2 dBm and −3.8 dBm, respectively.

**Figure 13:**
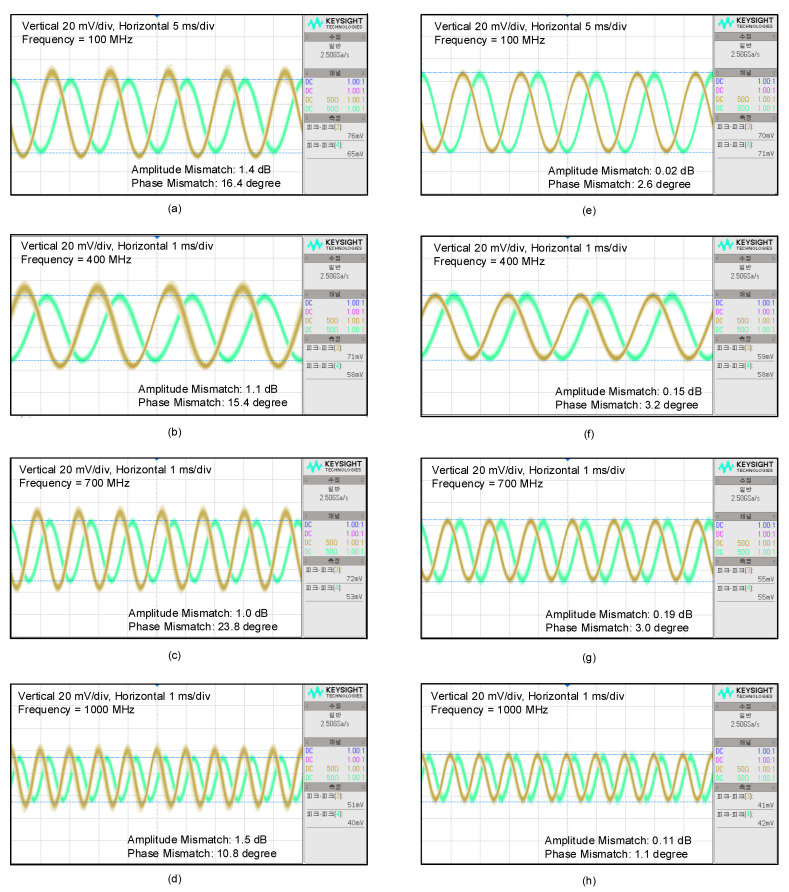
Measured I/Q baseband waveforms before and after calibration. (**a**) 100 MHz before calibration; (**b**) 400 MHz before calibration; (**c**) 700 MHz before calibration; (**d**) 1000 MHz before calibration; (**e**) 100 MHz after calibration; (**f**) 400 MHz after calibration; (**g**) 700 MHz after calibration; (**h**) 1000 MHz after calibration.

**Figure 14:**
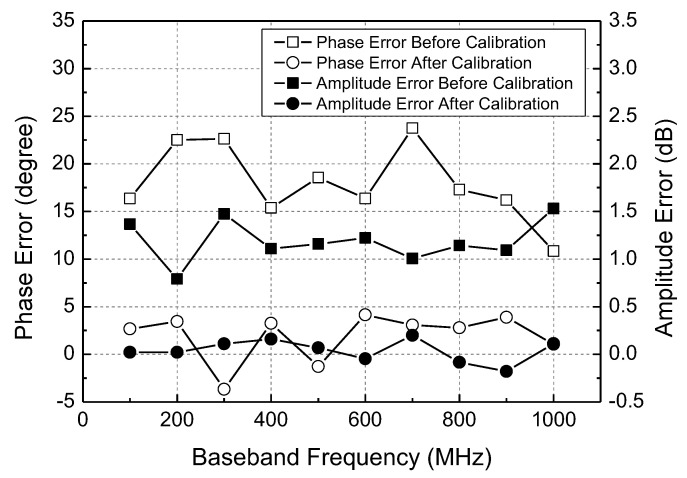
Measured I/Q amplitude and phase mismatches before and after calibration.

The proposed I/Q calibration circuit is tested by examining the I/Q baseband waveforms. The LO frequency is fixed at 23.5 GHz, and the RF frequency is tuned from 23.6 to 24.5 GHz to produce the I/Q baseband signal at 100–1000 MHz. The baseband waveforms before calibration are shown in Figure 13a–d for 100, 400, 700, 1000 MHz, respectively. The optimally calibrated conditions are obtained by carefully tuning the mixer’s gate bias control code and the LO buffer’s capacitance control code. The overall calibration process is carried out in the order that the amplitude mismatch is first calibrated through the mixer gate bias tuning, and the phase mismatch is then calibrated through the LO buffer tuning. The resulting baseband waveforms after calibration are shown in Figure 13e–h. It is clearly seen that the I/Q calibration circuit successfully minimizes the amplitude and phase mismatches. The measured mismatches before and after calibration are re-drawn in Figure 14. Across the baseband frequency from 100 to 1000 MHz, the uncalibrated phase mismatches of 10.8–23.8 degrees are significantly improved to 1.1–3.2 degrees after calibration, and the uncalibrated amplitude mismatches of 1.0–1.5 dB are also significantly improved to 0.02–0.19 dB after calibration. Note that the above calibration experiments are simply conducted by manually adjusting the calibration code while observing the baseband waveforms with bare eyes. Thus, we can expect that the calibrated mismatches could be much further improved if the calibration is performed by monitoring the IRR more closely with an aid of a separate digital signal-processing function block.

Table 1 summarizes the performances of this work and compares them to previous works. All of them are based on the same quadrature direct-conversion architecture. Nevertheless, the frequency band and intended application slightly varies, such as [10] for 35 GHz radar application, [8,9,21] for 24 GHz radar application, and [11] for 28 GHz 5G applications.

Note that [8,9,10] did not present any I/Q mismatch calibration techniques, whereas [21] presented only a phase mismatch calibration with a very narrow calibration range of only 4 degrees. [11] presented the I/Q mismatch calibration, while their phase calibration technique using QVCO should not be effective when a single-phase VCO is used. The I/Q calibration of this work is based on the mixer’s gate bias control for the amplitude tuning, and the LO buffer’s capacitance control for the phase tuning. The proposed gate bias control circuit for the amplitude mismatch calibration can be found in more conventional sub-6 GHz RF receivers such as 0.9/1.9 GHz cellular receiver [26], 2.4 GHz Bluetooth receiver [27], and 400 MHz MedRadio receiver [28]. In addition, the same technique was also proven effective for the 28 GHz millimeter-wave RF transmitter [2]. However, as mentioned earlier in Section 3.2, in spite of the similarities of this work and [2], the different design aspects induced by the difference of the RC load in the down-conversion mixer and the RLC load in the up-conversion mixer should be carefully taken into account for this mixer gate bias control technique. We have shown that the mixer gate bias control technique simultaneously induces amplitude and phase changes, hence the companion phase tuning circuit should be designed to sufficiently cover the total phase variation range. The measurement results show that the proposed calibration technique covers a wide range of mismatches with sufficiently fine resolution.

## 5. Conclusions

We have presented a CMOS quadrature direct-conversion RF receiver with wide-range and precise I/Q mismatch calibration for 24 GHz radar sensor applications. As a useful design guide for the I/Q mismatch calibration, the analytic relation of $\theta_{e.deg}=6.6\cdot a_{e.dB}$ is derived to show the equivalent individual contributions of the phase and amplitude mismatches to the IRR. The gate bias tuning method in the Gilbert-cell mixer is found to induce the amplitude and phase changes when it has a resistive load for a down-conversion mixer, whereas it induces only amplitude change and insignificant phase change when it has an inductive load for the up-conversion mixer. The proposed I/Q calibration circuit employs the mixer’s gate bias voltage control technique for the amplitude calibration, and the LO buffer’s varactor capacitor control technique for the phase calibration. The proposed calibration technique is shown to provide a sufficient precision and cover a wide tuning range. The prototype RF receiver-integrated circuit is fabricated in 65 nm RF CMOS process and successfully demonstrates the I/Q mismatch calibration performances.

## Figures and Tables

**Table 1:** Performance summary and comparison.

	This Work	[10]	[9]	[8]	[21]	[11]
RF Receiver Architecture	Direct Conversion	Direct Conversion	Direct Conversion	Direct Conversion	Direct Conversion	Direct Conversion
RF Frequency (GHz)	24	35	24	24	24	28
I/Q Amplitude Mismatch Calibration	Mixer Gate Bias Control	none	none	none	none	BBA Gain Control
I/Q Phase Mismatch Calibration	LO Buffer Cap Control	none	none	none	LO Buffer Cap Control	QVCO Tank Cap Control
Gain (dB)	31.5	33	36.7	31.5	24	69
IP_1dB_ (dBm)	−35.2	−23	−29.7	−24	−20	−68.9
Noise Figure (dB)	4.8	5.9–7.5	6.1	6.7	7.8	6.7
CMOS Process	65 nm	65 nm	130 nm	65 nm	180 nm	28 nm
